# Mucinous cystic neoplasms of the pancreas associated with pregnancy: Two case reports

**DOI:** 10.1097/MD.0000000000021471

**Published:** 2020-07-31

**Authors:** Fernando Revoredo, José de Vinatea, Gustavo Reaño, Luis Villanueva, Fritz Kometter, José Arenas, Patricio M. Polanco

**Affiliations:** a Department of General Surgery; b Department of Pathology, Hospital Nacional Guillermo Almenara Irigoyen, Lima, Perú; c Department of Surgery, UT Southwestern Medical Center, Dallas, TX.

**Keywords:** mucinous cystic neoplasms, pancreas, pregnancy

## Abstract

**Rationale:**

Although rare, pancreatic neoplasms can occur during pregnancy, both in benign and malignant forms. Mucinous cystic neoplasms (MCNs) of the pancreas, a type of these neoplasms, are precursor lesions to invasive pancreatic cancer. The presence of the ovarian-type stroma is a defining feature.

**Patient concerns:**

The first case was a 38-year-old woman in her 18th week of pregnancy with abdominal pain that worsens a few weeks later. The second case was a 30-year-old woman in her 17th week of pregnancy with abdominal pain in the left hypochondrium.

**Diagnosis:**

The patients were under clinical examination and laboratory test including carbohydrate antigen 19-9 (CA 19-9) and carcinoembryonic antigen (CEA). Both patients had magnetic resonance imaging (MRI). The diagnosis of a MCNs of the pancreas was done preoperatively in the 2 cases.

**Interventions:**

Both patients underwent distal pancreatectomy during pregnancy. One of them was an emergency laparotomy because of a ruptured MCN.

**Outcomes:**

Both patients were completely recovered from distal pancreatectomy and continued to full term, delivering a healthy baby by Caesarean section. After 6 years of follow-up, the first patient underwent a total gastrectomy, because of a gastric cancer with carcinomatosis. Currently the 2 patients are still alive after 8 years and 5 years of follow-up, respectively.

**Lessons:**

Surgical resection of MCNs during pregnancy should be considered during the second trimester given common distal pancreas location, rapid growth, risk of spontaneous rupture, and malignant potential.

## 1 Introduction

Mucinous cystic neoplasms (MCNs) are mucin-producing epithelial neoplasms of the pancreas and are precursor lesions to invasive pancreatic cancer that usually do not communicate with the pancreatic ductal system.^[1,2]^ Histologically, the MCNs have 2 distinct components: an inner epithelial layer and an ovarian-type sub-epithelial stroma.^[1–3]^ The epithelial layer is composed of tall, columnar, mucin-producing cells. The underlying ovarian-type stroma consists of densely packed spindle-shaped cells with round or elongated nuclei and sparse cytoplasm.^[2]^ The presence of the ovarian-type stroma is a defining feature of MCN and its presence has become a requirement for diagnosis.^[1–6]^

MCNs are relatively rare, accounting for around 8% of all surgically resected cystic neoplasms of the pancreas,^[2]^ and the vast majority of MCNs (95%) occur in woman.^[1,2,3,7]^ The female-to-male ratio is 20:1.^[2]^ The mean age at diagnosis is between 40 and 50 years with a range of 14 to 95 years.^[2,7]^ Most MCNs are located in the body and tail of the pancreas (95–98%).^[1,2,7]^

MCNs are classified according to the grade of epithelial layer dysplasia. Noninvasive MCNs are categorized as low-grade (adenoma), intermediate-grade (borderline), or high-grade dysplasia (carcinoma in situ). If there is a component of invasive carcinoma, the neoplasms are designated as MCNs with an associated invasive carcinoma.^[2,6]^ MCNs with invasive carcinoma frequently contain areas of low-grade, intermediate-grade, or high-grade dysplasia. These findings suggest that benign MCNs may progress to malignancy (an “adenoma to carcinoma” sequence over time).^[3]^ When an MCN evolves into invasive carcinoma, it is typically a tubular adenocarcinoma and rarely evolves into a colloid carcinoma or undifferentiated carcinoma with osteoclast-like giant cells.^[4]^ After surgical resection, in the absence of an associated invasive carcinoma, prognosis for MCN cases is excellent, with a 5-year survival rate of 100%, and in patients with an associated invasive carcinoma, the 5-year survival rate is 20% to 75%.^[1,3,6–9]^ MCNs with an associated invasive carcinoma appear to be fairly aggressive but have a better prognosis than ordinary ductal adenocarcinoma arising from pancreatic intraepithelial neoplasia, which show only a 10% to 15% 5-year survival. A postoperative surveillance is not mandatory for noninvasive MCNs, and for most patients with the disease, complete resection means curative therapy.^[1]^ In contrast, follow-up after resection for an MCN with an associated invasive carcinoma should be like that for pancreatic ductal adenocarcinoma.^[1]^

Pancreatic neoplasms, both benign and malignant, are uncommon during pregnancy.^[10]^ There have been some cases of pancreatic adenocarcinoma, pancreatic neuroendocrine tumors, and pancreatic cystic neoplasms in pregnant patients,^[10,11]^ which lead to dilemmas in diagnosis, management, and timing of surgical treatment.^[10]^ MCNs are the most frequently reported pancreatic neoplasms during pregnancy. It appears that MCNs developing during pregnancy show a different growth pattern^[11]^ and tend to be large.

Here, we report 2 new cases of MCNs of the pancreas that were diagnosed and managed during pregnancy and we carry out a review of all the MCN cases reported in association with pregnancy.

## 2 Patient selection and methods

The database of the Pancreas, Spleen, and Retroperitoneum Surgery Service at Hospital Nacional Guillermo Almenara Irigoyen in Lima, Peru, was reviewed. Patients with a histopathological diagnosis of MCN who underwent pancreatectomy from January 2009 to December 2018 were identified. Patients with MCN and pregnancy were selected. The presence of an ovarian-type stroma was a requirement for the diagnosis of MCN. The ovarian-type stroma was defined as a collection of densely packed spindle-shaped cells with sparse cytoplasm and round or elongated nuclei underlying the epithelium.^[2]^ The neoplasms were categorized according to the epithelial dysplasia as MCNs with low-grade, intermediate-grade, or high-grade dysplasia or as MCNs with an associated invasive carcinoma.^[2,11,14]^ In MCNs with low-grade dysplasia, the epithelial layer has minimal-to-mild architectural and cytological atypia with a slight increase in the size of basally located nuclei; mitoses are absent.^[2]^ MCNs with intermediate-grade dysplasia have mild-to-moderate architectural and cytological atypia with papillary projections or crypt-like invaginations, cellular pseudostratification caused by the crowding of slightly enlarged nuclei, and occasional mitoses.^[2]^ MCNs with high-grade dysplasia are characterized by significant architectural and cytological atypia, with the formation of papillae with irregular branching and budding, nuclear stratification with loss of polarity, pleomorphism, and prominent nucleoli. Mitoses are frequent and can be atypical.^[2]^ The definition of malignancy was reserved just for patients with an associated invasive carcinoma.^[2,6]^ The clinical records were reviewed to obtain clinical data, including age, gestational age at the time of diagnosis, neoplasm growth during pregnancy, maximum diameter of neoplasm (via computed tomography scan (CT scan) or magnetic resonance imaging (MRI) value), neoplasm location in pancreas, timing of surgery, spontaneous neoplasm rupture, surgical procedure performed, histopathological diagnosis, the presence of ovarian-type stroma estrogen receptors (ERs) and progesterone receptors (PRs), and data regarding follow-up. We defined the normal value for serum levels of tumor marker carcinoembryonic antigen (CEA) as <5 ng/mL, carbohydrate antigen 19-9 (CA 19-9) as <37 U/mL, and carbohydrate antigen 125 (CA 125) as <21 U/mL. The study was approved by the Hospital Nacional Guillermo Almenara Irigoyen institutional review board. The 2 patients have provided informed consent for publication of the cases. None of these cases have been previously reported.

## 3 Case reports

### 3.1 Case 1

**Figure 1:**
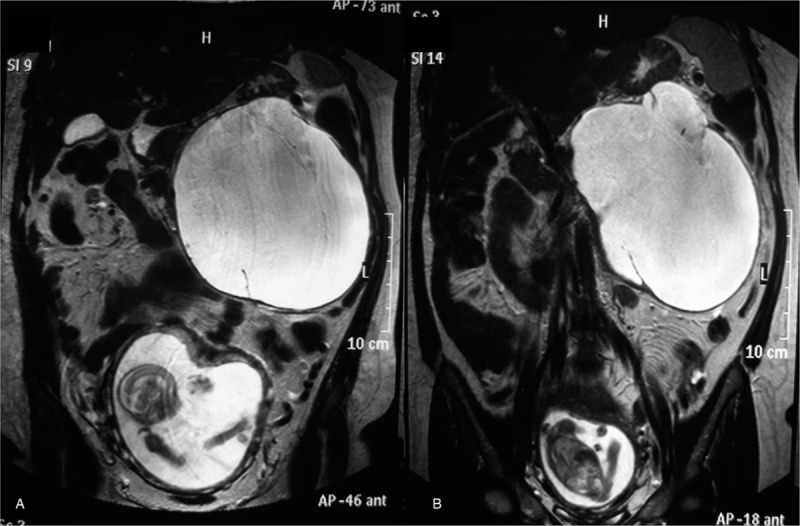
Magnetic resonance imaging showing a 20 cm × 18 cm × 18 cm cystic neoplasm arising from the pancreatic tail during pregnancy.

**Figure 2:**
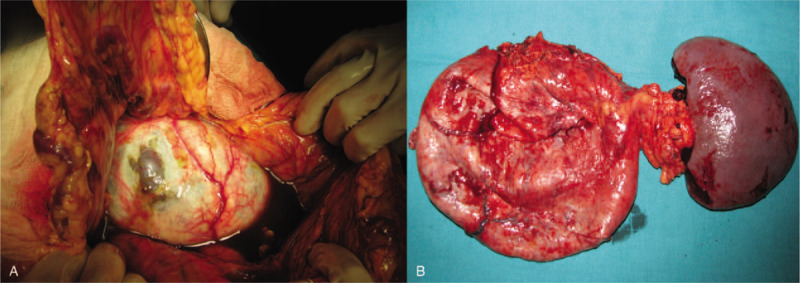
Surgical findings. (A) A ruptured pancreatic cystic neoplasm with dark-brownish fluid in the pancreatic bed. (B) A 20-cm-diameter thick-walled neoplasm, arising from the body and tail of the pancreas.

**Figure 3:**
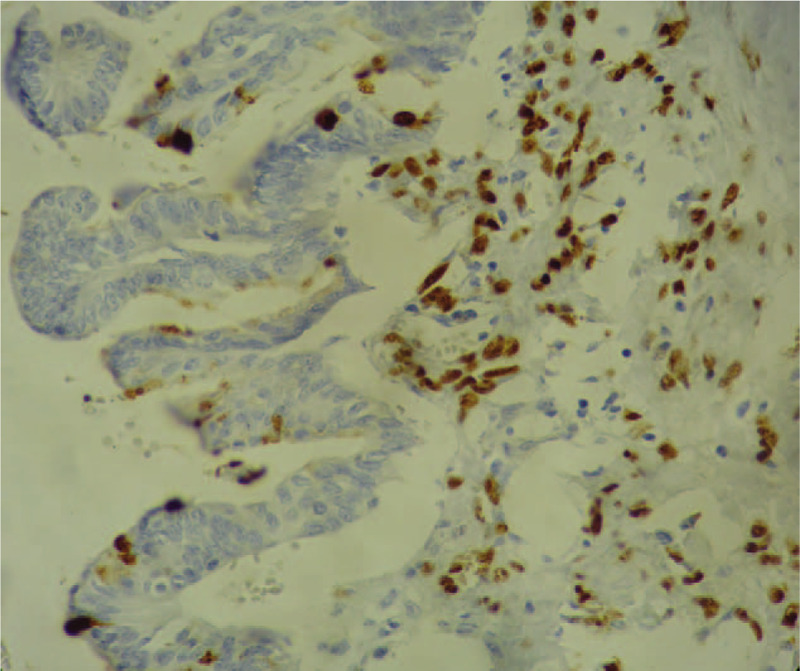
Immunohistochemical examination revealing ovarian-type stromal cells expressing the progesterone receptor.

A 38-year-old woman, gravida 3, para 2, presented in her 18th week of pregnancy with abdominal pain. An abdominal ultrasound showed a cystic mass of 17 cm × 5 cm × 13 cm in the left upper quadrant, with a volume of 1890 mL. The cystic mass had a thin wall with internal septations. An obstetric ultrasound showed a 17-week normal fetus. The serum level of tumor marker CEA was 0.91 ng/mL, CA 19-9 was 10.7 U/mL, and CA 125 was 18 U/mL. The ELISA for *Echinococcus* was negative. All other blood, serum, and urinary laboratory determinations were normal. An MRI revealed a 20 cm × 18 cm × 18 cm cystic neoplasm arising from the pancreatic tail (Fig. 1). An MCN of the pancreas was diagnosed. The patient was informed about the risk factors of her condition: malignant potential, rapid growth, the rupture of the neoplasm, and/or intrauterine growth restriction, but she refused the surgical treatment and expressed her strong desire to preserve the pregnancy until fetal maturation. The patient remained hospitalized at the obstetrics service, and at her 29th week she started having acute abdominal pain and tachycardia. A physical examination showed a decreased abdominal size and loss of strength. The patient was scheduled for an emergency laparotomy with the diagnosis of a ruptured MCN. The surgical findings included 1 L of a dark-brownish fluid in the pancreatic bed and a 20-cm diameter thick-walled neoplasm arising from the body and tail of the pancreas. It had a rupture of around 1 cm long in the thinner wall area. Some segmentary portal hypertension was found. A distal pancreatectomy with splenectomy was performed (Fig. 2). The patient was discharged 7 days postoperatively without complications. Pathological examination showed a mucinous neoplasm of the pancreas lined by columnar epithelial cells with an intermediate-grade of dysplasia (borderline), and positivity for CEA, with underlying ovarian-type stroma with positivity for PR and negativity for ER (Fig. 3). She continued to full term, delivering a healthy baby by Caesarean section at 41 weeks.

Six years later, during follow-up, the patient began having a progressive abdominal pain located in the epigastrium. This pain was worsened by meals and diminished with rest. It was also associated with early fullness, nausea, melena, and weight loss. After being managed for gastritis and after multiple endoscopies, she underwent a gastric biopsy that showed fragments of fibrous and muscular tissue infiltrated by well-differentiated tubular adenocarcinoma. A medical board was performed, and the patient underwent a laparotomy. A gastric cancer with carcinomatosis was found, then a palliative total gastrectomy was performed. Pathological examination showed a well-differentiated, infiltrating tubular gastric adenocarcinoma. The neoplasm infiltrated the serosa, subserosa, muscularis propia, and the mucosa (focally). Perineural invasion was present. Currently, 8 years of follow-up after the distal pancreatectomy, the patient is alive and receives irregular chemotherapy (capecitabine) because of her lack of adherence to the chemotherapy scheme.

### 3.2 Case 2

**Figure 4:**
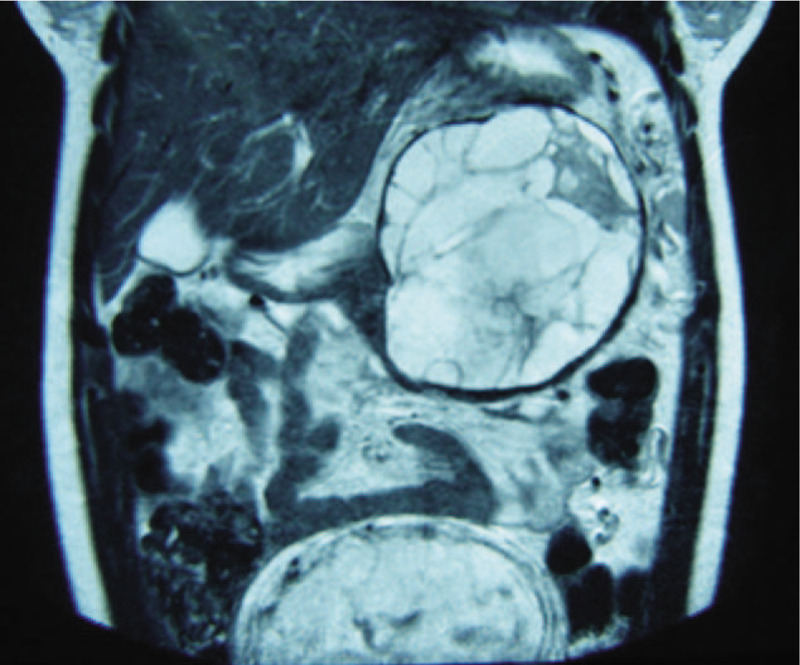
Magnetic resonance imaging showing an 11.8 cm × 11.6 cm × 9.5 cm multi-lobed neoplasm, arising from the body and tail of the pancreas with thin internal septations.

**Figure 5:**
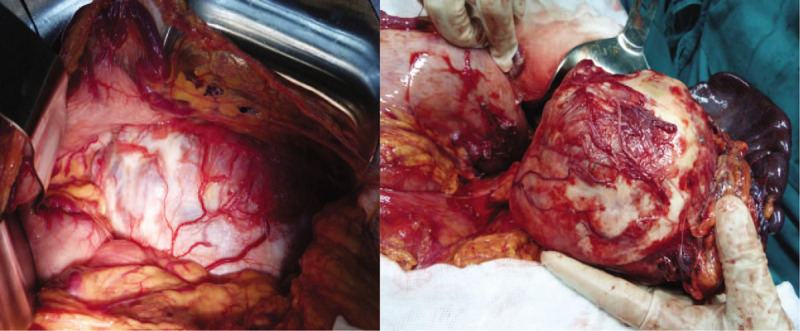
Surgical findings. A thick-walled, 15-cm-diameter cystic neoplasm arising from the pancreatic body and tail.

**Figure 6:**
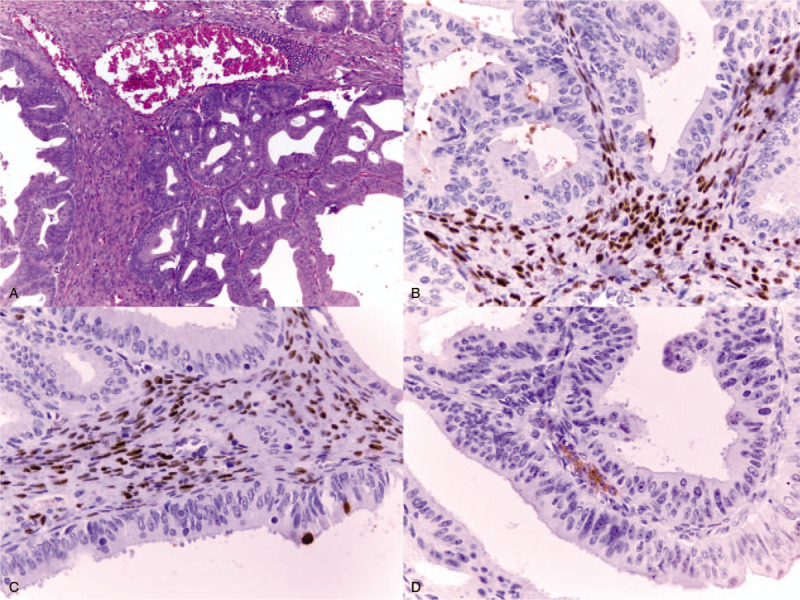
(A) The ovarian-type stroma (H&E staining, 4× magnification). Immunohistochemical examination revealed ovarian-type stromal cells expressing the estrogen receptor (B), progesterone receptor (C), and α-inhibin (D) (10×).

A 30-year-old woman, gravida 1, para 0, presented in her 17th week of pregnancy with intense abdominal pain located in the left hypochondrium. An abdominal ultrasound showed a multi-lobed cystic mass arising from the pancreatic body and tail. An obstetric ultrasound showed a normal fetus of 17 weeks and 5 days (via fetal biometry). An MRI revealed a multi-lobed neoplasm, arising from the body and tail of the pancreas with thin internal septations. The neoplasm measured 11.8 cm × 11.6 cm × 9.5 cm (Fig. 4). Serum level of tumor marker CEA was 51.92 ng/mL, CA 19-9 was 4.09 U/mL, and CA 125 was 38.6 U/mL. All other blood, serum, and urinary laboratory determinations were normal. A distal pancreatectomy with splenectomy was performed. The surgical findings included a 15-cm diameter thick-walled pancreatic neoplasm, with internal septations and dark-brownish mucinous fluid (Fig. 5). The neoplasm was adhered to the spleen, mesocolon, and diaphragm. Left-sided venous hypertension was noted because of splenic vein compression. The patient was discharged 12 days postoperatively without complications. Pathological examination showed a mucinous neoplasm of the pancreas lined by columnar epithelial cells with an associated invasive adenocarcinoma and extensive areas of high-grade dysplasia (carcinoma in situ). The underlying ovarian-type stroma showed positivity for PR, ER, and α-inhibin (Fig. 6). The patient refused to receive chemotherapy because of the concern for congenital malformations. She continued to full term, delivering a healthy baby by Caesarean section at 38 weeks. After delivery, the patient did not undergo chemotherapy and has remained symptom-free with no detectable recurrence for 5 years of follow-up. Her child is developing normally.

## 4 Discussion

There have been few reported cases of pancreatic neoplasms during pregnancy, the most common of which are MCNs. The size of this neoplasm could cause complications during pregnancy; this includes intrauterine growth restriction, compression of surrounding structures, pancreatitis, and neoplasm rupture.^[10]^

MCNs are lined by tall columnar epithelial cells that produce mucin,^[2,5]^ with varying degrees of architectural and cytological atypia. MCNs are classified as having low-grade dysplasia (72–87% of cases), intermediate-grade dysplasia (5–10%), high-grade dysplasia (5.5–13.4%), and associated invasive adenocarcinoma (3.8–36%).^[2,3,7,8,12–15]^ Identifying the underlying ovarian-type stroma is required for the diagnosis of MCN. The ovarian-type stroma expresses PR (60–90% of cases) and ER (30%). Luteinized cells, when present, label with antibodies against tyrosine hydroxilase, calretinin, and α-inhibin.^[2,5]^

The origin of ovarian-type stroma is still being debated. There are 2 hypotheses. The first is that these neoplasms arise from embryologic ovarian tissue deposited in the pancreas (ectopic ovarian stroma). This hypothesis is supported by the proximity of the left ovarian primordium to the dorsal pancreatic anlage during embryogenesis (4th–5th week). Moreover, dorsal pancreatic enlargement mainly affects the pancreatic body and tail. During that period, primordial ovarian cells could theoretically become incorporated into the pancreas, explaining the predilection of MCNs for the distal pancreas.^[7,16]^ The possible derivation of the stromal component of MCNs from the ovarian primordium is supported by morphology, the tendency to undergo luteinization, and immunophenotypical features. This ectopic ovarian stroma in the pancreas may release hormones and growth factors causing endodermally derived epithelium in its vicinity to proliferate and form cystic neoplasms. However, MCNs can arise in men, and none of these mechanisms account for this.^[5]^ The second hypothesis is that endodermally derived epithelium and primitive mesenchyme in the pancreas become hypersensitive to female sex hormone stimulation and start to proliferate.^[8]^ Regardless of its origin, it is clear that this stroma is hormone-sensitive; it is often admixed with luteal-type cells and it regularly expresses progesterone receptors,^[4]^ suggesting a hormone influence in the pathogenesis of these neoplasms.

In our literature review of 32 MCN cases during pregnancy,^[10,11,20–45]^ 30 neoplasms (93.8%) were located in the body/tail of the pancreas and 2 (6.2%) were in the head of the pancreas (Table 1). These findings are in accord with the 95% to 98% body/tail location reported for the no pregnant population.^[1,4,7]^

**Table 1:**
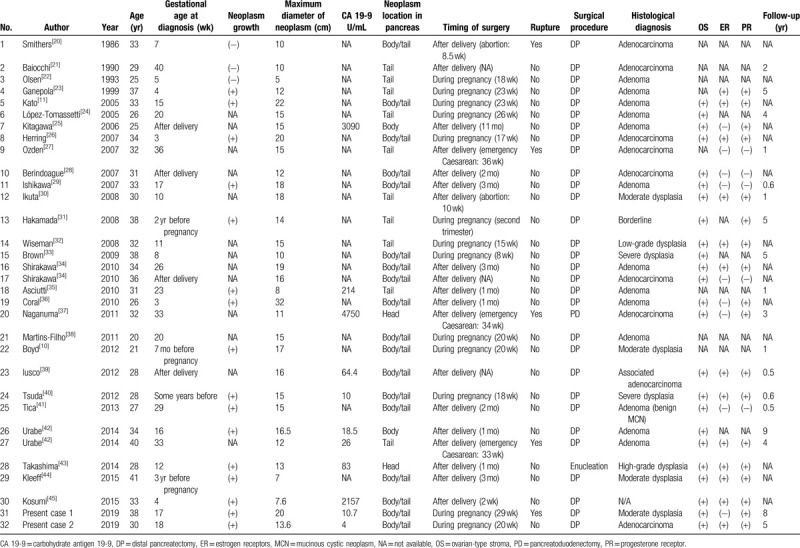
Clinicopathologic features of MCNs of pancreas associated with pregnancy. Each entry represents a single case report.

The preoperative diagnosis of MCN depends on a combination of clinical features, tumor markers, and imaging techniques such as CT, MRI, and endoscopic ultrasonography (EUS) with cyst fluid analysis.^[7]^ A single diagnostic CT is not associated with an increased risk of fetal malformations, although multiphase CT scans do increase the radiation exposure to the fetus and should be used sparingly.^[10]^ In addition, there may be an increased risk of spontaneous abortion associated with CT scanning within the first 2 weeks after conception and also a slightly increased risk of childhood cancers in an exposed fetus.^[10]^ Fetal teratogenicity and acoustic damage are the main concerns with MRI use during pregnancy, although several studies have failed to show adverse teratogenic, behavioral, or hearing effects.^[17]^ The principal advantage of MRI over ultrasonography and CT is the ability to image deep soft-tissue structures in a manner that is not operator-dependent and does not use ionizing radiation.^[17]^ The MRI is the imaging technique of choice for pregnant patients.^[10,17,18]^ The use of gadolinium contrast with MRI should be limited; it may be used only if it significantly improves diagnostic performance and is expected to improve the fetal or maternal outcome.^[18]^ After maternal administration, gadolinium appears rapidly in the fetal bladder and then is excreted into the amniotic fluid where it can be potentially swallowed by the fetus and absorbed from the fetal gastrointestinal tract.^[17]^

On cross-sectional imaging, an MCN appears as a well-capsulated, unilocular, or multilocular septated cystic lesion. The neoplasm is round to oval with a smooth external margin, and the wall of the cyst is typically thick with delayed enhancement. Peripheral calcification is seen in 10% to 25% of cases and is an important characteristic of the MCN that can be used to distinguish it from serous cystadenoma, which tends to have central calcification.^[19]^ MCNs usually do not communicate with the main pancreatic duct, a characteristic feature of the intraductal papillary mucinous neoplasm.^[6,19]^ When rarely present, this communication is due to fistula formation between the MCN and the pancreatic duct, rather than a true intraductal origin.^[8]^ Different attenuations or signal intensities may be noted within the cystic cavity, depending on whether mucoid or hemorrhagic fluid is present.^[19]^ A thickened wall with peripheral calcification, papillary proliferations, vascular involvement, hypervascular pattern, the presence of mural nodules, and lesion size (>6 cm) should be considered as suggestive of an MCN with malignant possibility.^[1,4,5]^

The EUS, with or without fine needle aspiration (FNA), is a diagnostic tool used in the workup for cystic neoplasms of the pancreas. EUS is particularly valuable in assessing diagnostic features and potential predictors of malignancy. The use of EUS-FNA varies widely throughout the world. An elevated CEA in the pancreatic cyst fluid is a marker that distinguishes mucinous from nonmucinous cysts, but not benign from malignant cysts. A cut-off of 192 ng/mL is 80% accurate for the diagnosis of a mucinous cyst. Cytology can be diagnostic, although the sensitivity is limited by scant cellularity; because of that, cytology is still considered investigational and should be performed only in centers with expertise in interpretation.^[6]^ A gastrointestinal endoscopy in pregnant patients is inherently risky because the fetus is particularly sensitive to maternal hypoxia and hypotension. Maternal oversedation resulting in hypoventilation or hypotension, or maternal positioning that precipitates inferior vena cava compression by the gravid uterus, can lead to decreased uterine blood flow and fetal hypoxia.^[46]^ Despite the above-mentioned risks and the limited evidence, a EUS-FNA could be performed during pregnancy, with close involvement of obstetrical staff to assist with management, which includes determining the degree of maternal and fetal monitoring; the patient should be in the lateral decubitus position before, during, and after the procedure; and if deep sedation is needed, it should be administrated by an anesthesia provider.^[46]^ Within the 32 case reports of MCNs associated with pregnancy, EUS was performed in 3 patients (9%),^[26,35,40]^ and just 1 patient had CEA in the pancreatic cyst fluid, with a value of 837 ng/mL.^[26]^ None of the 3 patients had morbidity related to the procedure.

If we analyze the 32 published case reports of MCNs associated with pregnancy,^[10,11,20–45]^ we find that the average size of the neoplasm is 14.5 cm, much larger than those reported for general population that range between 4.9 and 6.5 cm.^[3,8,9]^ The rapid growth of neoplasms during pregnancy^[10,11,23,26,29,31,35,36,40–45]^ suggests a possible relationship with female sex hormones.^[44]^ An interesting fact that strengthens this hypothesis is the finding reported by Tanaka et al^[47]^ of an MCN developing during continuous hormone replacement therapy after hysterectomy. There is no doubt that pregnancy triggers high levels of estrogens and progesterone. How sex hormones influence MCN growth and development is not currently known. Specifically, it is not clear if high levels of estrogen and progesterone may induce the proliferation of the stromal compartment, or may result in changes in the stromal cells that indirectly stimulate the growth of the neoplastic epithelial cells.^[44]^ The stromal component is not only a supporting tissue for the epithelium, but also critically involved in directing growth and differentiation. During neoplasm development, the stroma provides the extracellular matrix as an anchorage for the neoplasm cells. Stroma-derived growth as well as interactions between neoplastic cells and the extracellular matrix can play a role in both neoplasm cell migration and proliferation. The extracellular matrix may also function as a reservoir for growth factors.^[47]^

Excluding the case reports of high-grade dysplasia, we found 9 MCN cases (28%) with an associated invasive carcinoma, a rate much higher than that reported for the no pregnant population, which ranges from 3.9% to 16.3%.^[3,8,9,13,14]^ It is not known whether hormonal changes^[44]^ and the rapid neoplasm growth during pregnancy^[37]^ increases the rate of transformation of a benign neoplasm into a malignant one.^[44]^

Except in patients with a surgical contraindication, resection of MCNs is routinely proposed.^[2,6]^ For this reason, the natural history of unresected MCNs is poorly known.^[15]^ MCNs are usually located in the body/tail of the pancreas, and because of that, the most common surgical procedure performed for resecting these neoplasms is the distal pancreatectomy, which is a safe procedure in high-volume centers^[3]^ with an overall morbidity ranging from 10% to 30% and a mortality rate of almost 0%.^[1]^ Within the 32 case reports of MCNs associated with pregnancy, 30 patients (93.8%) underwent distal pancreatectomy, 1 (3.1%) underwent pancreatoduodenectomy, and 1 (3.1%) underwent enucleation. Thirteen patients (40%) underwent surgery during pregnancy, and of these, 11 underwent surgery during the second trimester, 1 during the first trimester, and 1 during the third trimester (Table 1). There were no major obstetric complications reported in the 13 patients who underwent surgery during pregnancy. Non-obstetric and non-urgent surgery during pregnancy should be performed in the second trimester when preterm contractions and spontaneous abortion are least likely.^[48]^

Among the 32 case reports of MCNs associated with pregnancy, 5 cases (15%) were spontaneously ruptured. This condition forced an emergency Caesarean section in 4 cases.^[20,27,37,42]^ In our own first case report in this article, in spite of the neoplasm spontaneously rupturing and an emergency distal pancreatectomy, the patient continued to full term, delivering by Caesarean section at 41 weeks. Three (60%) of the five cases in which MCNs spontaneously ruptured during pregnancy had an associated invasive carcinoma.^[20,27,37]^ Within the greatest series of MCNs, there are no reports of spontaneously ruptured MCNs,^[3,8,13]^ although there are at least 15 other case reports of MCNs spontaneously rupturing without an association with pregnancy and requiring surgery or some other kind of emergency procedure.^[49–63]^ Of these, 6 cases (40%) had an associated invasive carcinoma.^[49,52,57,58,60,61]^ Even though there are not published reports of ruptured MCNs showing the relationship with malignant transformation, based on the above findings, we could hypothesize that the spontaneous rupture of an MCN expresses a malignant behavior.

In our patient with spontaneous rupture, histopathological analysis found an MCN with intermediate dysplasia; nevertheless, 6 years later the patient was diagnosed with an advanced gastric cancer (carcinomatosis), and she underwent a palliative total gastrectomy. The histopathology showed a well-differentiated tubular adenocarcinoma. The neoplasm infiltrated the serosa, muscularis propia, submucosa, and just focally the mucosa, meaning that the neoplasm seemingly involved from outside to inside. This finding suggests that perhaps there was a local recurrence that infiltrated the stomach. Hakamada et al^[31]^ reported a case of a 38-year-old female, in whom a 10 cm pancreatic cyst was pointed out during her first pregnancy. She refused surgery and delivered her baby uneventfully. During her second pregnancy, and because of progressive anemia, hematemesis, and tarry stools, she underwent urgent distal pancreatectomy, splenectomy, and a partial resection of the gastric wall where the tumor perforated. The postoperative course was uneventful, and she delivered her second baby. A diagnosis of borderline (intermediate-grade dysplasia) MCN was made. At the site of gastric perforation, no neoplastic change was identified. Nine months after surgery, a 3-cm neoplasm near the pancreatic stump was found. Following the diagnosis of a local recurrence of the MCN, the patient underwent an en-bloc resection of the pancreatic stump, gastric wall, and left adrenal gland. The pathology of the pancreatic area was a borderline (intermediate-grade dysplasia) MCN. On the other hand, the area of the gastric wall contained an anaplastic carcinoma with osteoclastoid giant cells, atypical spindle-shaped cells, and round cells, showing sarcomatous changes. These findings make us suppose that once the neoplastic cells were seeding either in a viscera or the peritoneum, they will continue with the adenoma–carcinoma sequence described for MCNs.^[3]^

Fernandez del Castillo et al^[64]^ reported that a serum CA19-9 concentration of >37 U/mL had a positive predictive value of 95% for malignant or potentially malignant lesions, with a sensitivity of only 35.8%. Park et al^[9]^ and Jang et al^[13]^ found that serum CA 19-9 was significantly elevated in MCNs with invasive carcinoma. On the other hand, in a recent series, Postlewait et al^[65]^ found that an elevated serum CA 19-9 was associated with increased risk of malignancy; however, this association did not persist in a multivariable analysis. In reports of MCNs associated with pregnancy, CA 19-9 levels were recorded in just 11 cases, and of these, 3 were remarkable and elevated in the presence of a noninvasive MCN, with values ranging from 213.7 U/mL to 3090 U/mL.^[25,35,45]^ In 2 patients^[37,39]^ with MCNs and an associated invasive carcinoma, the CA 19-9 was elevated (64 U/mL and 4750 U/mL). In our second case report, the CA 19-9 was normal; however, the final diagnosis was of an MCN with an associated invasive carcinoma. These conflicting findings may be because CA 19-9 values were not available in 21 patients (65%).

No conclusive data exist for neoadjuvant or adjuvant therapy for MCN with an associated invasive carcinoma. Current treatment options have been extrapolated from the management of pancreatic ductal adenocarcinoma and malignant intraductal papillary mucinous neoplasms, and usually include gemcitabine or fluorouracil.^[66,67]^ The combination of gemcitabine–oxaliplatin has been proposed to be more effective in terms of clinical progression-free survival.^[68]^ FOLFIRINOX (folinic acid, fluorouracil, irinotecan, and oxaliplatin) has been most effective for metastatic pancreatic cancer with a median overall survival of 11 months.^[69]^ The use of FOLFIRINOX in the management of malignant MCNs has already been reported.^[70]^ Systemic treatment with chemotherapy during the first trimester of pregnancy is associated with a high risk of miscarriage and in some cases congenital malformations reaching as high as 20%, this being the period of organogenesis.^[71]^ In patients requiring chemotherapy initiation during the first trimester, pregnancy termination would be considered.^[71]^ Administration of chemotherapy during the second and third trimesters has not been associated with significant fetal defects in the short or long term.^[71,72]^ When it is possible to delay the initiation of chemotherapy beyond the 14th week, the risk of severe problems for the fetus are low and pregnancy termination is not needed.^[72]^ The recommendations are mainly based on large case series of breast, cervical, ovarian, lymphoma, and lung cancer patients.^[71,72]^ In pancreatic cancer, there are at least 2 case reports of chemotherapy during pregnancy.^[10,73]^ The first report by Boyd et al^[10]^ described the application of two 4-week cycles of gemcitabine in a 37-year-old pregnant woman, starting at week 24 of pregnancy as adjuvant therapy after the resection of pancreatic adenocarcinoma. A healthy child was born in the 34th week. The patient died 1 year after surgical resection. Further follow-up of the infant was not reported.^[10]^ The second case report by Lubner et al^[73]^ described the application of 2 cycles of gemcitabine in a 37-year-old pregnant female, beginning at her 24th week of pregnancy until the 31st week. Labor was induced at 35 weeks and she delivered a male infant. The patient died 12 months after the diagnosis and the child met all appropriate developmental milestones in terms of growth, cognitive development, language development, and socialization (2-year follow-up).^[73]^ Within the 9 cases of MCNs during pregnancy with an associated invasive carcinoma, just 2 received adjuvant chemotherapy (gemcitabine).^[27,37]^ In both cases the chemotherapy was after delivery.

## 5 Conclusion

Pancreatic cystic neoplasms during pregnancy are infrequent, and of these, MCNs are the most frequent. MRI is the method of choice for the diagnosis of an MCN in pregnant women. MCNs during pregnancy should be managed with surgical resection when possible, during the second trimester because of the malignant potential, rapid growth, and risk of spontaneous rupture of the neoplasm. A distal pancreatectomy during pregnancy has not been associated to major obstetric morbidity. The spontaneous rupture of an MCN would express a malignant behavior.

## Acknowledgments

The author would like to thank Dave Primm for help in editing this article.

## Author contributions

**Conceptualization:** Fernando Revoredo, José de Vinatea, Gustavo Reaño, Luis Villanueva, Fritz Kometter, José Arenas, Patricio Polanco.

**Data curation:** Fernando Revoredo, José de Vinatea, Gustavo Reaño, Luis Villanueva, Fritz Kometter, José Arenas, Patricio Polanco.

**Formal analysis:** Fernando Revoredo, José de Vinatea, Gustavo Reaño, Luis Villanueva, Fritz Kometter, José Arenas, Patricio Polanco.

**Investigation:** Fernando Revoredo, José de Vinatea, Gustavo Reaño, Luis Villanueva.

**Methodology:** Fernando Revoredo, José de Vinatea, Gustavo Reaño.

**Project administration:** Fernando Revoredo.

**Resources:** Fernando Revoredo, José de Vinatea, Gustavo Reaño.

**Validation:** José Arenas.

**Visualization:** Fernando Revoredo, José de Vinatea, Gustavo Reaño, Luis Villanueva, Fritz Kometter, José Arenas, Patricio Polanco.

**Writing – original draft:** Fernando Revoredo, José de Vinatea, Gustavo Reaño, Luis Villanueva, Fritz Kometter, José Arenas, Patricio Polanco.

**Writing – review & editing:** Fernando Revoredo, José de Vinatea, Gustavo Reaño, Fritz Kometter, José Arenas, Patricio Polanco.
